# Inborn errors of human B cell development, differentiation, and function

**DOI:** 10.1084/jem.20221105

**Published:** 2023-06-05

**Authors:** Stuart G. Tangye, Tina Nguyen, Elissa K. Deenick, Vanessa L. Bryant, Cindy S. Ma

**Affiliations:** 1 Garvan Institute of Medical Research, Darlinghurst, Australia; 2 School of Clinical Medicine, Faculty of Medicine and Health, UNSW Sydney, Sydney, Australia; 3 Immunology Division, Walter & Eliza Hall Institute, Parkville, Australia; 4 Department of Medical Biology, University of Melbourne, Parkville, Australia; 5 Department of Clinical Immunology & Allergy, The Royal Melbourne Hospital, Parkville, Australia

## Abstract

B cells develop from hematopoietic stem cells in the bone marrow. Once generated, they serve multiple roles in immune regulation and host defense. However, their most important function is producing antibodies (Ab) that efficiently clear invading pathogens. This is achieved by generating memory B cells that rapidly respond to subsequent Ag exposure, and plasma cells (PCs) that continually secrete Ab. These B cell subsets maintain humoral immunity and host protection against recurrent infections for extended periods of time. Thus, the generation of antigen (Ag)-specific memory cells and PCs underlies long-lived serological immunity, contributing to the success of most vaccines. Our understanding of immunity is often derived from animal models. However, analysis of individuals with monogenic defects that disrupt immune cell function are unprecedented models to link genotypes to clinical phenotypes, establish mechanisms of disease pathogenesis, and elucidate critical pathways for immune cell development and differentiation. Here, we review fundamental breakthroughs in unraveling the complexities of humoral immunity in humans that have come from the discovery of inborn errors disrupting B cell function.

## Teaser

The fundamental importance of the role of human B cells in host defense against infectious diseases has been established by the discovery of inborn errors of immunity that disrupt B cell development, differentiation, or function. These findings have laid the groundwork for understanding mechanisms of disease and revealing pathways to improve humoral immunity and treat disease.

## Introduction

### B cell development and function

**Figure 1.**
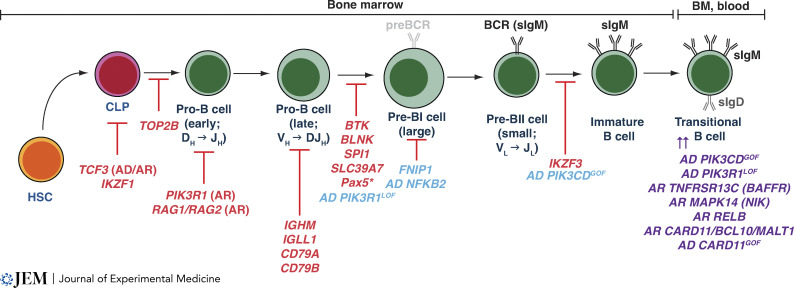
**Intrinsic molecular requirements for human B cell development as revealed by IEIs.** B cell development occurs in the BM and involves the sequential progression of HSCs into CLP, which then gives rise to progenitor B cells committed to a B cell fate. B cell development requires assembly and expression of a functional BCR. The initial stages of Ig gene rearrangement occur at the early and late pro-B cell stages. Pro-B cells that successfully express cytoplasmic Igµ chains develop into pre-BI cells that express a preBCR; rearrangement of Ig L chain genes occurs in pre-BII cells, which then express a functional sIgM molecule. Pre-B cells develop into immature B cells which then give rise to transitional B cells which egress from the BM and enter the peripheral circulation. Genetic variants causing severe disruption to B cell development are shown in red, and those having a milder effect on B cell development are shown in blue. Some of these latter variants also result in an accumulation of transitional B cells (shown in purple). Data for the impact of Pax5 deficiency on B cell development are inferred from studies of a mouse model expressing the human mutations.

B cells develop from hematopoietic stem cells (HSCs) in the bone marrow (BM) through a series of iterations whereby HSCs give rise to common lymphoid progenitors (CLP), which develop into progenitor (pro), precursor (pre), and then immature B cells (Morgan and Tergaonkar, 2022; Pieper et al., 2013; Uckun, 1990; Fig. 1). Each of these stages is characterized by the sequential and temporal rearrangement of genes encoding V, D, and J elements of the IgH chain, acquisition of expression of a pre-B cell receptor (BCR) complex comprising IgH chain and a surrogate IgL chain on large pre-B cells, followed by recombination of genes encoding an IgL chain in small pre-B cells, and then finally expression of a functional BCR on immature B cells.

Rearrangement of genes encoding the V, D, and J elements of the IgH (VDJ) and IgL (VJ) chains during B cell development is regulated by the coordinated induction and activity of the recombination activating genes *RAG1* and *RAG2* in pro-(VDJ) and pre-(VJ) B cells (Morgan and Tergaonkar, 2022; Pieper et al., 2013; Uckun, 1990). As VDJ recombination is a random process, some immature B cells that express a BCR recognizing endogenous self-antigens (Ag) will inevitably be generated. To establish B cell tolerance and avoid autoimmunity, developing B cells are screened for self-reactivity. Thus, immature B cells that bind self-Ag with sufficient avidity undergo either receptor editing, which results in expression of a BCR that does not recognize self-Ags, or clonal deletion resulting in elimination of autoreactive B cells (Pelanda et al., 2022; Pieper et al., 2013). Immature B cells that do not bind self-Ag, or bind self-Ag with minimal avidity, then exit the BM as transitional B cells, and undergo final maturation and selection to yield a pool of naive B cells capable of recognizing a potentially unlimited number and array of foreign Ags (Fig. 1; Morgan and Tergaonkar, 2022; Pelanda et al., 2022; Pieper et al., 2013; Uckun, 1990).

### Naive B cells differentiate into memory cells and plasma cells (PCs) during germinal center (GC) reactions

**Figure 2.**
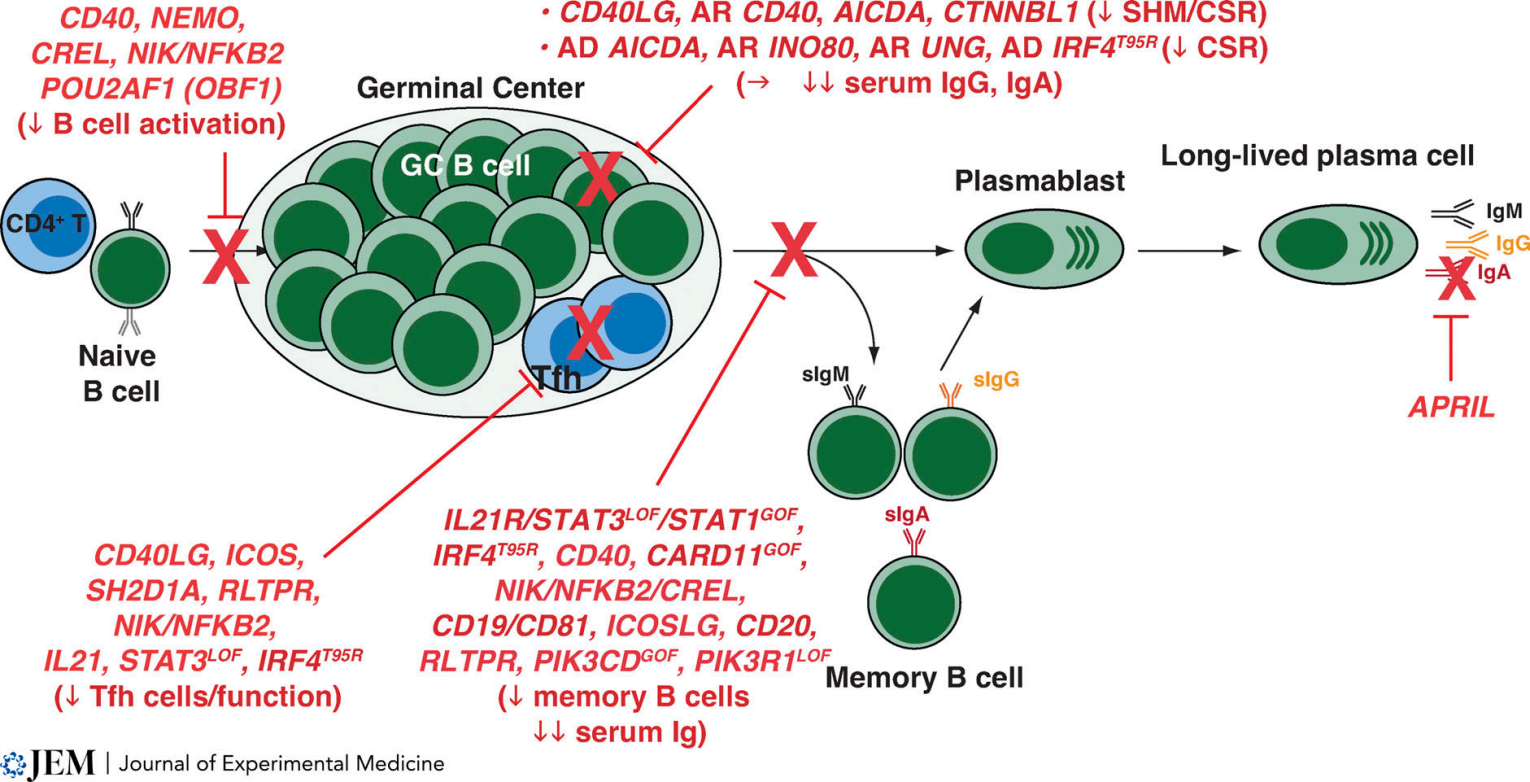
**IEIs can disrupt human B cell differentiation by intrinsic and extrinsic mechanisms.** In secondary lymphoid tissues, Ag-specific naive B cells interact with cognate CD4^+^ T cells and seed a GC. Here, B cells undergo intense proliferation (clonal expansion) and SHM. GC B cells with the highest affinity for Ag compete for survival signals provided by Tfh cells and then differentiate into memory or PCs that produce high-affinity neutralizing Ig (IgM, IgG, and IgA). IEIs affecting various stages of TD B cell differentiation are shown in red.

When naive B cells traffic through secondary lymphoid tissues and encounter foreign Ag, they differentiate into various fates depending on signals provided by the microenvironment. T-independent and T-dependent (TD) Ag can induce naive B cells to rapidly differentiate into short-lived plasmablasts that predominantly secrete IgM and preferentially localize to extrafollicular regions of lymphoid tissues. These cells provide initial protection against pathogen infection (Tangye and Tarlinton, 2009). Activated B cells can also undergo Ig class switch recombination (CSR) to express IgG, IgA, or IgE, and seed GCs, which form transiently in lymphoid tissues. Within GCs, Ag-specific B cells undergo clonal expansion and somatic hypermutation (SHM) of IgV genes. On one hand, GC B cells that acquire the highest affinity for specific Ag compete for limited survival signals provided by CD4^+^ T follicular helper (Tfh) cells—CD4^+^ T cells specialized to support B cell differentiation—dendritic cells and follicular dendritic cells generate memory B cells or PCs (Fig. 2; Victora and Nussenzweig, 2022). On the other hand, B cells that acquire self-reactivity as a result of SHM, or retain low-affinity BCRs, undergo apoptosis and are purged from the B cell repertoire (Victora and Nussenzweig, 2022). Once generated, memory cells and PCs migrate to lymphoid tissues, peripheral blood, or the BM (Tangye and Tarlinton, 2009; Fig. 2). The anatomical distribution of PCs and memory cells enables the immune system to provide basal protection against infection by producing antibodies (Ab) that clear invading pathogens, and responding rapidly following re-encounter with specific pathogens to mitigate infectious diseases (Robinson et al., 2020; Tangye and Tarlinton, 2009).

### Inborn errors of immunity (IEIs) reveal the fundamental roles of B cells in host defense

IEIs result from monogenic germline variants and are characterized by defects in immune cell development and/or function (Tangye et al., 2022). Due to an immune-deficient state, most affected individuals are susceptible to severe, recurrent, and/or potentially fatal infections. The critical role of humoral immunity in host defense is evident from IEIs that impact B cells (Pieper et al., 2013; Tangye et al., 2022). A classic example is the description by Colonel Ogden Bruton in 1952 of the first case of agammaglobulinemia in an 8-yr-old boy who experienced 19 episodes of severe bacterial infection; importantly, infusion of donor immune serum prevented further infections (Bruton, 1952). These observations established that agammaglobulinemia caused recurrent infections, and serum gammaglobulin contained Ab capable of preventing infection. It cannot be emphasized enough that these observations in a single patient not only pre-dated by 4 yr the serendipitous finding by Glick and colleagues that the Bursa of Fabricius was required for generating Ab responses in chickens (Glick et al., 1956), and the formal discovery of B cells by Max Cooper by >10 yr (Cooper et al., 1965), but also led to the findings that:

• X-linked agammaglobulinemia (XLA) is caused by mutations in *BTK* (Conley et al., 2009);
• Bruton’s tyrosine kinase (BTK) links the BCR to many intracellular signaling pathways and is critical for human B cell development and function;
• Ab deficiency can be treated by Ig replacement therapy irrespective of genetic cause; and
• Pharmacological targeting of BTK would revolutionize treatment for B cell malignancies and some autoimmune conditions decades later (Garg et al., 2022; Gayko et al., 2015).

**Figure 3.**
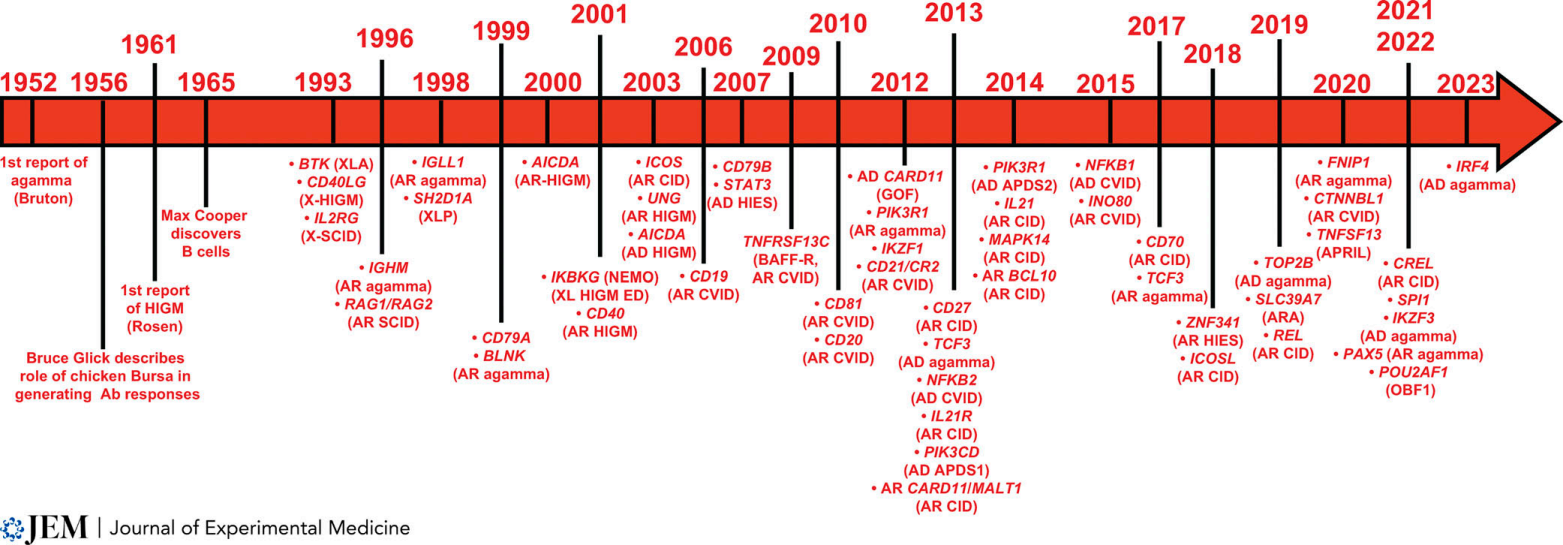
**Timeline of key discoveries of IEI affecting human B cells.** Key discoveries over the last 70 yr that have advanced the field of human B cell biology. agamma, agammaglobuliemia; AD, autosomal dominant; AR, autosomal recessive; HIES, hyper IgE syndrome; HIGM, hyper IgM syndrome; XL-HIGM ED, X-linked hyper IgM syndrome with ectodermal dysplasia; X-SCID, X-linked severe combined immunodeficiency; XLP, X-linked lymphoproliferative disease.

Such insights, often gleaned from single patients, kindreds, or small numbers of affected individuals, are frequently repeated such that our understanding of human B cell biology has been greatly enriched by seminal discoveries of IEI that impact B cells (Fig. 3).

### Agammaglobulinemia and B cell deficiency

#### BTK mutations cause XLA

Bruton’s description of congenital agammaglobulinemia is generally acknowledged as being one of the first reports of an IEI (Bruton, 1952; Conley et al., 2009; Cooper and Lawton, 1972). The first genetic etiology of agammaglobulinemia (i.e., early-onset immunodeficiency, repeated hospitalization for severe life-threatening bacterial infections, <1% peripheral blood B cells, severe reduction of all serum Ig) was identified in 1993 as variants in *BTK*, an X-linked gene (Tsukada et al., 1993; Vetrie et al., 1993; Fig. 3). Thus, BTK deficiency causes XLA and accounts for ∼85% of agammaglobulinemia cases (Conley et al., 2009).

BTK deficiency arrests development at the pro-B cell stage (Fig. 1), causing a 100–1,000-fold reduction in recirculating B cells, and a lack of all serum Ig isotypes (Cooper and Lawton, 1972; Conley et al., 2009; Dobbs et al., 2011). The few B cells that can be detected in peripheral blood of XLA patients exhibit a phenotype of immature transitional B cells, characterized by elevated expression of IgM, reduced expression of CD19 and CD21, and BCRs enriched for autoreactive specificities (Conley, 1985; Dobbs et al., 2011; Ng et al., 2004; Suryani et al., 2010). Female carriers of *BTK* mutations are healthy. Furthermore, their B cells exhibit skewed inactivation of the X chromosome harboring the mutant *BTK* allele (Conley et al., 1986). This indicates that impaired or absent BTK function causes an intrinsic survival disadvantage in developing B cells. Thus, BTK is necessary for generating a pool of mature B cells capable of responding to foreign Ag to provide Ab-mediated host defense against infection pathogens. Interestingly, a number of cases have been reported of males who presented with recurrent bacterial infections, hypogammaglobulinemia or Ig subclass deficiencies and variable numbers of circulating B cells, and were found to have hypomorphic variants in *BTK* that had modest effects on B cell development and differentiation (Geier et al., 2018; Krüger et al., 2020; Mitsuiki et al., 2015; Toker et al., 2022). This highlights the importance of validating variants in genes that are known to cause disease and identified in individuals with atypical presentations of classic IEIs.

#### Phenocopies of XLA: Elucidation of molecular causes of autosomal recessive (AR) agammaglobulinemia

**Figure 4.**
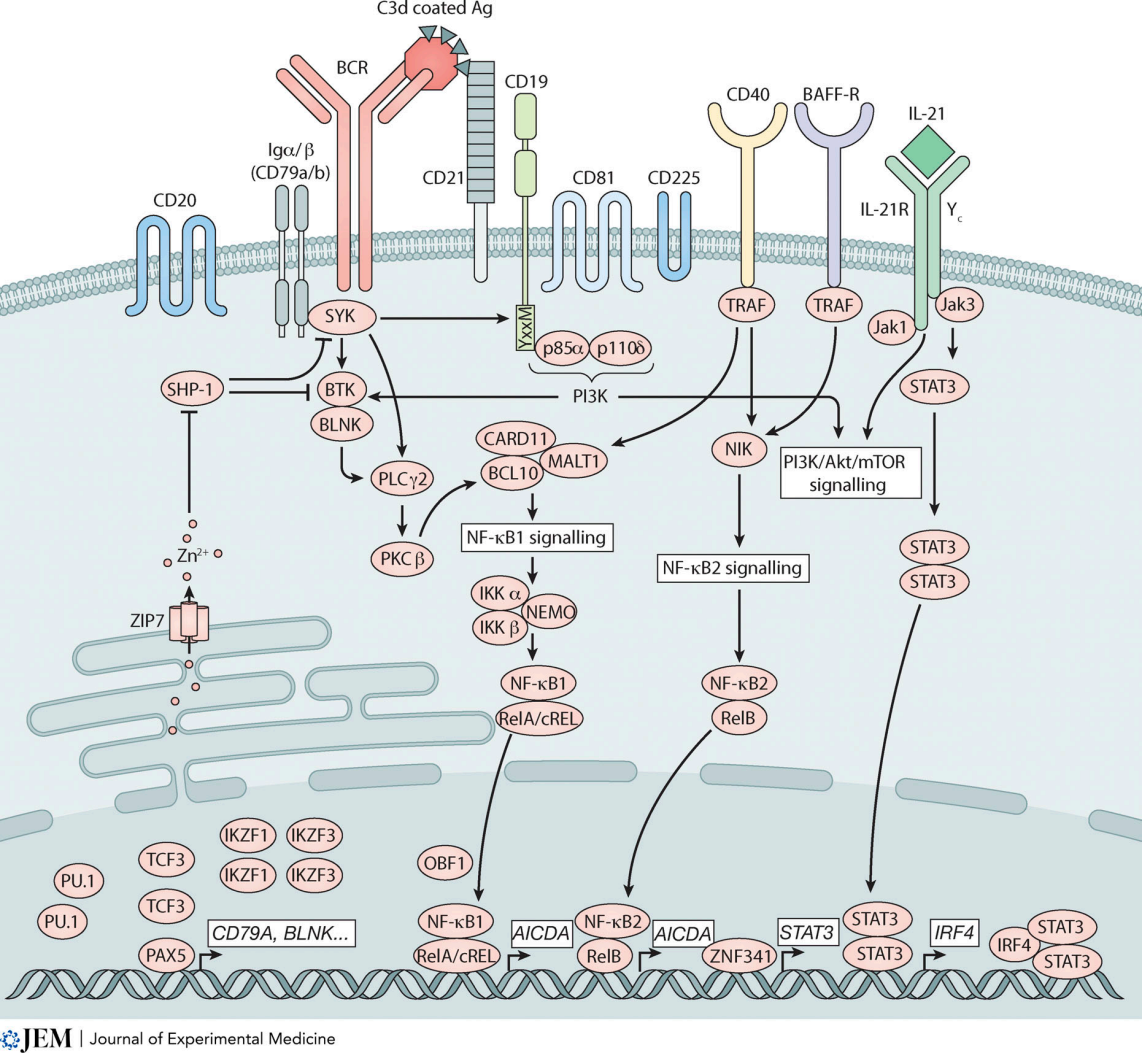
**Intracellular signaling pathways and transcriptional networks necessary for human B cell development and differentiation.** Diagrammatic representation of the key receptor signaling pathways, intermediates, and transcription factors that cooperatively underpin human B cell development, differentiation, and effector function.

Subsequent studies of cases resembling XLA identified biallelic mutations in *IGHM* (Igµ chain; Yel et al., 1996), *IGLL1* (λ5 surrogate L chain; Minegishi et al., 1998), *CD79A* (Igα; Minegishi et al., 1999a), *CD79B* (Igβ; Dobbs et al., 2007; Ferrari et al., 2007), *BLNK* (Minegishi et al., 1999b), *PIK3R1* (Conley et al., 2012; Tang et al., 2017), or *PIK3CD* (Sogkas et al., 2018; Fig. 3). Igµ, λ5, Igα, and Igβ comprise the pre-BCR expressed during B cell development. *PIK3R1* and *PIK3CD* encode the p85 regulatory and p110δ catalytic subunits of PI3K, which is activated following BCR ligation to generate phosphatidylinositol 3,4,5-triphosphate (PIP3). PIP3 binds to and recruits BTK to the cell membrane to undergo autophosphorylation-mediated activation, interacts with BLNK, and then phosphorylates specific substrates (Fig. 4; Conley et al., 2009; Niiro and Clark, 2002; Tangye et al., 2019). Collectively, these cases further highlighted the fundamental requirement for intact BCR signaling in human B cell development, and revealed critical, complementary, coordinated, and integrated roles of BTK, BLNK, and PI3K in this process (Figs. 1 and 4). Interestingly, individuals heterozygous for *BLNK*, *IGHM*, *IGLL1*, *CD79A*, or *CD79B* variants are healthy, indicating that haploinsufficiency or dominant negative (DN) functions of the encoded proteins are not pathogenic. Thus, B cell deficiency and agammaglobulinemia result from loss of expression or loss of function (LOF) of encoded proteins. As *BTK*, *BLNK*, *IGHM*, *IGLL1*, *CD79A*, and *CD79B* are prominently—or exclusively—expressed in B cells, T cell development and differentiation were largely unaffected in individuals with mutations in these genes.

B cell development is also severely disrupted by biallelic variants in either *RAG1* or *RAG2* (Fig. 1; Schwarz et al., 1996). These variants reduce the activity of RAG1 and RAG2 proteins, thus preventing rearrangement and recombination of IgH and IgL chains and subsequent assembly of a functional BCR complex (Fig. 1; Schwarz et al., 1996). However, unlike other IEI affecting B cell development resulting in agammaglobulinemia, variants in *RAG1* or *RAG2* cause severe combined immunodeficiency (SCID) because recombination of TCR chains during thymic development is also compromised.

#### Transcription factors required for B cell development

IEI also identified key transcription factors necessary for B cell lineage commitment. *SPI1* encodes PU.1 that functions at early stages of specification of HSC to the B cell lineage. *TCF3* encodes E12 and E47 that are critical for lymphoid progenitors to commit to the B lineage. Within hematopoietic cells, PAX5 is exclusively and stably expressed in B cells and is required for commitment of progenitor cells to the B lineage and progression of developing B cells beyond the pro-B cell stage. PAX5 initiates and maintains B cell commitment by repressing genes that influence HSC commitment to other lineages and promoting expression of genes crucial for B cell development (Nutt and Kee, 2007). Ikaros family members IKAROS (*IKZF1*), HELIOS (*IKZF2*), and AIOLOS (*IKZF3*) are highly expressed in leukocytes and form homo- and hetero-dimers to regulate gene expression and hematopoiesis (Yamashita and Morio, 2021). Recently, individuals with B lymphopenia, agammaglobulinemia, and recurrent sinopulmonary infections due to mutations in *TCF3*, *SPI1*, *PAX5*, *IKZF1*, or *IKZF3* have been reported (Fig. 3).

*TCF3.* Five unrelated individuals with <1% blood B cells and dramatic reductions in B-lineage BM cells due to a loss of CLPs had heterozygous DN mutations in *TCF3* (Al Sheikh et al., 2021; Boisson et al., 2013; Dobbs et al., 2011). Biallelic nonsense *TCF3* variants were also identified in two individuals with B cell deficiency and hypo/agammaglobulinemia (Ben-Ali et al., 2017). In this latter setting, heterozygous carriers of nonsense *TCF3* variants were healthy, indicating E2A haploinsufficiency does not impact B cell development (Figs. 1 and 3).

*SPI1.* Six unrelated individuals with heterozygous mutations in *SPI1* had 0–0.2% blood B cells. Pro-B cells were the only B cells detected in their BM (Le Coz et al., 2021), consistent with PU.1 expression increasing as B cells transit through the pro → pre stages of development (Nutt and Kee, 2007; Fig. 1). PU-1–deficient pro-B cells expressed reduced levels of genes required for B cell development compared to PU.1-sufficient pro-B cells (*IGLL5*, *IGHM*, *CD79B*, and *BTK*; Le Coz et al., 2021). Notably, mutant PU.1 did not affect WT PU.1 function, indicating the mechanism of pathogenicity is PU.1 haploinsufficiency. Thus, human B cell development is strictly dependent on PU.1 dose, as one WT *SPI1* allele could not support B cell development in affected individuals (Le Coz et al., 2021).

*PAX5.* A single individual with compound heterozygous *PAX5* variants had reduced levels of all serum Ig isotypes and peripheral B-lymphopenia (Kaiser et al., 2022). Mice expressing patient-specific *PAX5* alleles (*Pax5*^R31Q/E242^*) had an accumulation of pro-B cells and 5–10-fold fewer pre- and immature B cells in BM compared to WT mice (Fig. 1). *Pax5*^R31Q/E242^* mice also had significantly reduced peripheral B cells and levels of serum IgG (10–20-fold), IgA (∼5-fold), and IgM (2-fold). Mechanistically, *Pax5*^*R31Q*^ encoded a hypomorphic protein, impaired in regulating 10–20% of PAX5 target genes (Kaiser et al., 2022). Thus, human PAX5 deficiency blocks B cell development resulting in B cell cytopenia, hypogammaglobulinemia, and impaired Ab responses.

*IKZF1.* The first case of *IKZF1* mutation was reported in 2012 in an infant with pancytopenia, including a near absence of B cells (Fig. 3; Goldman et al., 2012). Since then, heterozygous *IKZF1* mutations have been reported in ∼100 individuals. B cell lymphopenia, hypogammaglobulinemia, and recurrent infections are highly penetrant clinical features of most patients with *IKZF1* mutations (Hoshino et al., 2017; Kuehn et al., 2022; Yamashita and Morio, 2021). IKAROS deficiency disrupts B cell development by impairing the generation of CLP and B-lineage cells in the BM (Fig. 1; Hoshino et al., 2017; Kuehn et al., 2022; Kuehn et al., 2016; Yamashita and Morio, 2021). Notably, the block in B cell development is quantitative rather than qualitative as proportions of developing B cell subsets in IKAROS deficiency were largely normal (Hoshino et al., 2017; Kuehn et al., 2016). Thus, *IKZF1* mutations predominantly impact the trajectory of CLP to the B cell lineage (Fig. 1).

*IKZF3.* Three related individuals with recurrent sinopulmonary infections, undetectable blood B cells, and very few BM B cells had a heterozygous *IKZF3* variant (AIOLOS^G159R^; Yamashita et al., 2021). Unlike IKAROS, AIOLOS is absent from CLP and early B cell progenitors but is highly expressed in pre-B cells (Yamashita et al., 2021). Analysis of mice bearing the patient-specific allele (*Ikzf3*^+/G159R^) revealed a block at the pre-B cell stage (Fig. 1; Yamashita et al., 2021). Importantly, *Ikzf3*^+/G159R^ intrinsically affected murine B cell development (Yamashita et al., 2021). Thus, *IKZF3* variants cause B cell lymphopenia by disrupting progression of pre-B to immature B cells, rather than impacting the progression of CLP into B-lineage precursors.

AIOLOS^G159R^ was LOF for binding canonical DNA sequences defined for WT AIOLOS, gain of function (GOF)/neomorphic for binding novel motifs, and impaired WT AIOLOS from binding target sequences by DN. Furthermore, AIOLOS^G159R^/IKAROS heterodimers exhibited altered chromatin localization and DNA binding. Thus, AIOLOS^G159R^ compromised B cell development by LOF, GOF, DN, and impeding IKAROS function (Yamashita et al., 2021; Yamashita and Morio, 2021). This provides an explanation for the similarity in clinical features of IKAROS and AIOLOS deficiency.

Combined, novel IEIs that intrinsically disrupt human B cell development identified key transcriptional regulators with non-redundant roles in commitment of CLP to a B cell fate or regulating expression of key genes required for B cell development (Figs. 1 and 3).

#### Additional genetic causes of agammaglobulinemia and B cell deficiency

*SLC39A7.* The obligate role of divalent cations in signaling pathways is well-established (Vaeth and Feske, 2018). For instance, mutations disrupting Mg^2+^ (*MAGT1*) or Ca^2+^ (*STIM1*, *ORAI1*) transport in lymphoid cells (Vaeth and Feske, 2018) cause combined immunodeficiencies (CID) predominantly due to defects in functions of T cells and NK cells, rather than B cells (Vaeth and Feske, 2018). Recently, the importance of zinc in B cell development was revealed by identifying seven individuals from six families with biallelic hypomorphic variants in *SLC39A7*, encoding the zinc transporter ZIP7 (Anzilotti et al., 2019; Erdős et al., 2022; Fig. 3).

All patients had recurrent bacterial infections, agammaglobulinemia, very few blood B cells, and a severe block at the pro → pre-stage of B cell development (Fig. 1; Anzilotti et al., 2019; Erdős et al., 2022). By generating mice carrying patient hypomorphic *Slc39a7* alleles, Anzilotti et al. (2019) elegantly determined the causes of the selective B cell deficiency due to impaired ZIP7 function. ZIP7^P198A/P198A^ mice recapitulated the severe reductions in BM and peripheral B cells, low levels of serum Ig, and an intrinsic block at the pro-B cell stage of development noted in affected humans (Fig. 1). Total phosphatase activity was increased in ZIP7^P198A/P198A^ B cells, dampening overall BCR signaling. Thus, consistent with zinc inhibiting phosphatases (Vaeth and Feske, 2018), hypomorphic ZIP7 reduces cytoplasmic zinc concentrations thereby enabling increased phosphatase activity which sufficiently attenuates BCR signaling to disrupt B cell development (Anzilotti et al., 2019). Notably, biallelic *SLC39A7* variants had no effect on T cell signaling, development, or function (Anzilotti et al., 2019). Thus, similar to mutations in genes encoding the BCR complex (*IGHM*, *IGLL1*, *CD79A*, and *CD79B*), downstream mediators of BCR signaling (*BTK*, *BLNK*, *PIK3R1*, and *PIK3CD*), or transcription factors that regulate some of these key genes (*SPIB* and *PAX5*), ZIP7 deficiency abolishes early B cell development by impairing BCR function (Fig. 1).

*FNIP1.* Folliculin-interacting protein 1, encoded by *FNIP1*, interacts with folliculin to modulate AMP kinase activity, a sensor of energy consumption and regulator of cellular metabolism. Fnip1 deficiency in mice increased mTORC1 activity, mitochondrial biogenesis, and autophagy, disrupting B cell development and causing agammaglobulinemia (Siggs et al., 2016). To date, six patients from five families have been identified with biallelic *FNIP1* variants (Niehues et al., 2020; Saettini et al., 2021; Fig. 3). All patients had frank B cell deficiency, agammaglobulinemia, and recurrent respiratory infections (Niehues et al., 2020; Saettini et al., 2021). Limited analysis of BM samples suggested FNIP1 deficiency affects the early stages of B cell development, evidenced by increased proportions of pro-B and pre-B1 cells and reductions in pre-BII and immature B cells (Niehues et al., 2020; Saettini et al., 2021; Fig. 1). Molecular mechanisms underlying this selective effect of FNIP1 deficiency on early B cell development are unclear. However, increased mTOR and AMPK activity indicates perturbations to cellular energy homeostasis compromise B cell development and/or survival.

*TOP2B.* Topoisomerase 2B induces transient double-stranded DNA breaks to modulate topological chromatin conformation and maintain genomic integrity during replication; it also regulates transcription of particularly long genes (Austin et al., 2021). 11 individuals with B cell immunodeficiency, limb anomalies, and urogenital malformations, or Hoffman’s syndrome were found to have heterozygous *TOP2B* variants (Broderick et al., 2019; Erdős et al., 2021; Papapietro et al., 2020; Fig. 3). Despite variability in the impact of *TOP2B* variants on developmental features, a unifying trait of all patients was greatly reduced peripheral B cells, hypogammaglobulinemia, and recurrent respiratory infections (Broderick et al., 2019; Erdős et al., 2021; Papapietro et al., 2020). Analysis of BM from patients and mice harboring a patient mutation (*Top2b*^EE587E/WT^) identified a block at the CLP → pro-B stage of development (Fig. 1; Broderick et al., 2019; Papapietro et al., 2020).

*TOP2B* variants were LOF and DN (Broderick et al., 2019; Papapietro et al., 2020). Top2b is more highly expressed in B cells than T cells, and CLPs from *Top2b*^EE587E/WT^ mice expressed significantly lower levels of *Pax5* and *Foxo1*—factors important for B cell development (Nutt and Kee, 2007)—than *Top2b*^WT/WT^ CLPs (Broderick et al., 2019). This potentially explains why immune lineages other than B cells were unaffected (Broderick et al., 2019; Erdős et al., 2021; Papapietro et al., 2020). Thus, B cell development is highly sensitive to impaired TOP2B function. However, the exact molecular mechanism underlying selective B cell deficiency due to DN TOP2B and altered genomic integrity remains incompletely defined.

### Inborn errors affecting B cell differentiation and humoral immunity

In the 1960s and 1970s, pioneering clinical immunologists were harnessing emerging techniques (of the times!) to characterize different classes of serum gammaglobulins or lymphocytes in distinct immune deficiencies. These studies followed the seminal discoveries of XLA by Bruton in 1952 (Bruton, 1952), of the requirement for the Bursa of Fabricius in chickens to generate Ab responses by Bruce Glick in 1956 (Glick et al., 1956), and of B cells by Max Cooper in 1965 (Cooper et al., 1965; Fig. 3). Thus, it became possible to define immune-deficient states according to immune phenotype and serology. Rosen et al. (1961) described two males (Rosen et al., 1961) and one female (Rosen and Bougas, 1963) with recurrent bacterial infections and increased levels of 19S (i.e., modern day IgM) but lacked 7S (i.e., IgG, IgA) gammaglobulins. These probably represent the first reports of XL and AR hyper-IgM syndrome (Fig. 3; Fadlallah et al., 2020; Rosen and Bougas, 1963). Cooper et al. (1965) and Siegal et al. (1971) extended Rosen’s observation by reporting additional cases of “XL immunodeficiency with hyper-IgM syndrome” (HIGM) and likely cases of AR HIGM, as well as examples of “agammaglobulinemia with B cells” (Cooper and Lawton, 1972; Siegal et al., 1971). Over the past 30 yr, the genetic cause of many IEI presenting as severe infections despite intact B cell development has been identified (Fig. 3). These molecular lesions disrupt signaling pathways initiated following interactions between B cells, specific Ag, and cognate CD4^+^ T cells, resulting in impaired CSR, GC formation, SHM, memory B cell and PC generation, hypogammaglobulinemia, and/or functional Ab deficiency (Fig. 2).

#### Defects in the BCR/CD19 co-receptor complex

The BCR interacts with a complex comprising CD19, CD21, CD81, and CD225 (Fig. 4; Wentink et al., 2018). CD21 is the receptor for complement component C3d, and CD19 transduces signals following engagement of CD21; CD81 is required for CD19 expression. Ag-C3d aggregates the BCR with the CD19/CD21/CD81 complex, amplifying and sustaining BCR signaling. This also decreases the threshold for B cells to respond to limited Ag and bridges the adaptive and innate immune systems (Wentink et al., 2018).

Biallelic mutations in *CD19* (*n* = 10; Kanegane et al., 2007; Skendros et al., 2014; van Zelm et al., 2006; Vince et al., 2011; Wentink et al., 2018) or *CD81* (*n* = 2; van Zelm et al., 2010; Yang et al., 2022) cause severe recurrent respiratory tract infections, hypogammaglobulinemia (reduced IgG in all; low IgM and/or IgA in ∼50%), impaired vaccine responses, and reduced memory B cells; however, B cell development is intact (Fig. 2). Although GCs were detected in lymph nodes of CD19-deficient patients (van Zelm et al., 2006), SHM in IgV genes of patient B cells was reduced compared to healthy donors (Kanegane et al., 2007; van Zelm et al., 2014; van Zelm et al., 2006; van Zelm et al., 2010; Wentink et al., 2015). Thus, CD19 or CD81 deficiency impedes affinity maturation. Mutations in *CD81* abolished CD19 expression on patients’ B cells, indicating the requirement for CD81 in stabilizing CD19 expression, and explaining similarities in clinical features of these IEI (van Zelm et al., 2010; Yang et al., 2022). CD19 or CD81 deficiency reduced BCR-mediated signaling, proliferation, and differentiation in B cells from affected patients (van Zelm et al., 2006; van Zelm et al., 2010; Yang et al., 2022). *CD19* mutations also reduced CD21 expression on B cells (Kanegane et al., 2007; van Zelm et al., 2006; Vince et al., 2011), potentially further compromising function of the BCR/CD19 complex. Heterozygous carriers of *CD19* or *CD81* mutations are asymptomatic, establishing haploinsufficiency of these genes is not pathogenic.

In contrast to CD19 or CD81 deficiency, biallelic *CD21* (*CR2*) mutations causes a milder immunodeficiency: hypogammaglobulinemia, normal/mildly reduced serum IgM or IgA, fewer memory B cells, variable respiratory infections, and low-normal responses to protein and polysaccharide Ags (*n* = 4; Rosain et al., 2017; Thiel et al., 2012; Wentink et al., 2015). Responses of CD21-deficient B cells to an Ag-C3 complex were reduced, but direct BCR engagement induced intact B cell activation (Thiel et al., 2012). Furthermore, SHM in CD21-deficient memory B cells was only modestly reduced, and significantly greater than CD19-deficient B cells (Wentink et al., 2015). Thus, although CD19, CD81, and CD21 form a multimeric signaling complex with the BCR, CD19/CD81 and CD21 have unique roles in regulating human B cell responses. Specifically, CD21 deficiency can be compensated by signaling through receptors whose expression and function does not require CD21. CD19 or CD81 deficiency may also reflect effects of these mutations on expression/function of additional co-receptors—CD21—together with loss of CD19. These findings also highlight that CD19 has key roles in B cell responses beyond it functioning exclusively as the signal transduction partner of CD21.

The tetraspanin transmembrane molecule CD20 is another well-defined receptor expressed on B cells (Fig. 4); indeed, it is typically used to identify and enumerate human B cells and is the target of the biological therapeutic rituximab used to treat human B cell malignancies and some autoimmune conditions (LeBien and Tedder, 2008; Paley et al., 2017). Despite its ubiquitous expression during B cell development and maturation, the specific function of CD20 during humoral immune responses remains enigmatic. Some insights have been gleaned from the discovery of one individual with a homozygous *CD20* mutation (Kuijpers et al., 2010). Clinically, the patient experienced intermittent respiratory infections and persistently low levels of serum IgG and was diagnosed with common variable immunodeficiency (CVID) during her first decade of life. Numbers and proportions of B cells, titers and affinity of tetanus-specific IgG, levels of SHM, as well as an in vitro proliferation and calcium flux in response to BCR ligation and T-independent and TD stimuli were intact. However, proportions of class switched memory B cells, Ab responses to pneumococcal vaccination and in vitro production of IgG by activated B cells were reduced (Fig. 3; Kuijpers et al., 2010). It should be noted though that as the functional assays relied on responses of total B cells, then any reduction in IgG secretion noted in vitro could reflect the presence of fewer IgG^+^ memory B cells in the patient ex vivo compared to healthy donors. Despite this caveat, while CD20 appears to be dispensable for B cell development and responses to TD Ag, it appears to have a key role in regulating humoral immunity to T-independent Ags.

#### B cell–intrinsic defects directly impacting Ig class switching and SHM

A key component of an effective B cell response is CSR in naive B cells to express IgG, IgA, or IgE (de la Morena, 2016; Durandy et al., 2007; Notarangelo et al., 2006). IEIs intrinsically affecting CSR fall into two categories: those that disrupt signals inducing CSR and those that directly affect the molecular machinery required for this process. Engagement of CD40 on B cells by CD40L expressed on activated CD4^+^ T cell is a critical driver of CSR. Thus, the former category includes IEIs due to *CD40LG* (discussed further below) or *CD40* mutations. Since 2001, five patients have been identified with CD40 deficiency (Ferrari et al., 2001; Lougaris et al., 2005; Renner et al., 2021; Fig. 3). The original example of the second category was reported in 2000 as AR mutations in *AICDA* (Revy et al., 2000; Fig. 3), encoding activation-induced cytidine deaminase (AID) which is involved in CSR and SHM (de la Morena, 2016; Durandy et al., 2007; Notarangelo et al., 2006). Subsequently, patients were identified with biallelic mutations in other genes involved in CSR/SHM: *UNG* (*n* = 3; Imai et al., 2003), *INO80* (*n* = 2; Kracker et al., 2015), and *CTNNBL1* (*n* = 1; Kuhny et al., 2020; Fig. 3).

Patients with these B cell–intrinsic IEIs display similar phenotypes: recurrent infections, reduced serum IgA and IgG but normal/high IgM, impaired responses to vaccination, and a deficiency of class-switched memory B cells. However, there are several key clinical differences: CD40/CD40L deficiency results in severe opportunistic infections (due to defects in CD4^+^ T cell–mediated myeloid cell activation), while patients lacking AID, UNG, or CTNNBL1 exhibit follicular hypoplasia and autoimmunity (de la Morena, 2016; Ferrari et al., 2001; Imai et al., 2003; Kuhny et al., 2020; Lougaris et al., 2005; Notarangelo et al., 2006; Renner et al., 2021; Revy et al., 2000).

Interestingly, monoallelic *AICDA* variants that truncate the last 10–12 amino acids of AID protein have been identified in 18 patients from seven kindreds with a hyper-IgM-type phenotype (Durandy et al., 2007; Fadlallah et al., 2020; Imai et al., 2005; Kasahara et al., 2003; Kermode et al., 2022; Fig. 3). These patients presented with recurrent infection, increased levels of serum IgM, reduced serum IgG and IgA, and impaired Ab responses to vaccines and infection. However, whilst CSR was abolished in vivo and in vitro, SHM was unaffected in these patients (Durandy et al., 2007; Fadlallah et al., 2020; Imai et al., 2005; Kasahara et al., 2003; Kermode et al., 2022; Fig. 2). Patients also did not develop autoimmune features characteristic of AR AID deficiency (Durandy et al., 2007). Thus, AD *AICDA* variants affecting the C-terminal domain of AID can disrupt Ig CSR resulting in humoral immunodeficiency. This likely results from a DN effect of the truncated AID protein, rather than haploinsufficiency, because carriers of heterozygous *AICDA* variants—including null mutation—that are pathogenic in the homozygous state are healthy (Durandy et al., 2007; Fadlallah et al., 2020).

CD40 signaling in B cells initiates SHM and CSR via induction of AID. Thus, it is not surprising that these processes are severely affected by recessive mutations in *CD40* or *AICDA*, or XL *CD40LG* mutations (de la Morena, 2016; Notarangelo et al., 2006; Renner et al., 2021; van Zelm et al., 2014). Interestingly, while variants in *UNG*, *INO80,* and *CTNNBL1* all impact CSR, they do not universally affect SHM. Thus, *UNG* mutations alter the pattern, and possibly quality, but not overall level of SHM (de la Morena, 2016; Imai et al., 2003; Notarangelo et al., 2006), the *CTNNBL1* variant reduced but did not ablate SHM (Kuhny et al., 2020), while *INO80* deficiency had little effect on SHM (de la Morena, 2016; Kracker et al., 2015; Notarangelo et al., 2006). This suggests that the effector function of Ig acquired following CSR is critical to effective host defense even when B cells can undergo SHM and affinity maturation.

OBF-1 (also known as OCA-B or BOB1; encoded by *POU2AF1*) is a B cell–specific transcription factor originally considered to be important for regulating Ig transcripts during B cell development (Teitell, 2003). The generation of gene-targeted mice revealed that Obf-1 does have a modest role during B cell development, evidenced by two- to fourfold reductions in numbers of splenic B cells. However, the more striking function of OBF-1 was in governing the formation of GCs and establishing humoral immunity in murine models of viral infection or immunization (Teitell, 2003). Recently, the first case of human OBF-1 deficiency, due to homozygous variants in *POU2AF1*, was reported (Kury et al., 2021; Fig. 3). Despite normal numbers of peripheral B cells, the OBF-1–deficient patient presented with recurrent respiratory infection, agammaglobulinemia, and an absence of class switched memory B cells (Kury et al., 2021). Furthermore, OBF-1–deficient B cells exhibited impaired biochemical responses and reduced differentiation into IgG-expressing or plasmablast-type cells following in vitro stimulation with CD40L, TLR, or BCR agonists. Importantly, reconstitution of patient-derived OBF-1–deficient B cells with wild-type OBF-1 restored these functional defects, indicating that the *POU2AF1* variants identified are likely pathogenic (Kury et al., 2021). These findings highlight a critical role for OBF-1 in human B cell differentiation. As CD40 stimulation induces OBF-1 expression in B cells (Teitell, 2003), OBF-1 deficiency probably disrupts aspects of CD40-induced human B cell activation (Figs. 2 and 4).

#### B cell–extrinsic defects: Impaired generation and function of Tfh cells

Defective humoral immunity can result from B cell–extrinsic mechanisms due to mutations in genes predominantly expressed in T cells (Tangye et al., 2022; Tangye and Ma, 2021). XL HIGM is caused by hemizygous variants in *CD40LG* (Allen et al., 1993; Aruffo et al., 1993; DiSanto et al., 1993; Korthäuer et al., 1993; Ramesh et al., 1993). CD40L deficiency causes severe and frequent opportunistic infections (*Pneumocystis*, *Cryptosporidia*; de la Morena, 2016). ICOS deficiency causes recurrent bacterial, viral, and opportunistic infections, immune dysregulation and malignancy (Grimbacher et al., 2003; Schepp et al., 2017). The single case of IL-21 deficiency presented with recurrent sinopulmonary infections and early onset colitis (Salzer et al., 2014), while ∼50% of patients with XL lymphoproliferative disease due to *SH2D1A* mutations (encoding SAP) have recurrent infections and impaired vaccine responses (Ma et al., 2007).

These IEIs all share hypogammaglobulinemia, reduced IgM^+^ and class-switched memory B cells, few or poorly formed GCs, and diminished specific Ab responses (de la Morena, 2016; Ma et al., 2007; Renner et al., 2021; Salzer et al., 2014; Schepp et al., 2017; Warnatz et al., 2006). Interestingly, IgA^+^CD27^−^ memory B cells persist at normal numbers in CD40L deficiency (van Zelm et al., 2014). Importantly, in vitro function of B cells from these patients is intact (Ma et al., 2007; Ma et al., 2016; Mayer et al., 1986; Salzer et al., 2014), consistent with low or absent expression of *CD40L*, *IL21*, *ICOS*, or *SH2D1A* in human B cells. These findings indicated that impaired B cell differentiation results from compromised CD4^+^ T cell function, specifically Tfh cells. Tfh cells express high levels of CD40L, ICOS, and IL-21, which interact, respectively, with CD40, ICOS-ligand (ICOS-L), and IL-21R on B cells to mediate memory cell and PC generation (Tangye and Ma, 2021). SAP is also highly expressed by Tfh cells and signals downstream of SLAM family receptors on T cells (Ma et al., 2007). IEI due to mutations in *CD40* (Ferrari et al., 2001; Lougaris et al., 2005), *ICOSL* (Roussel et al., 2018), or *IL21R* (Cagdas et al., 2021; Kotlarz et al., 2013) phenocopy CD40L, ICOS, or IL-21 deficiencies (Fig. 2).

Patients with impaired CD40L/CD40, ICOS/ICOS-L, or IL-21/IL-21R signaling have reduced circulating Tfh (cTfh) cells, while SAP deficiency compromises Tfh cell function (Fig. 3; Tangye and Ma, 2021). cTfh cells are also quantitatively and qualitatively reduced by mutations in *CARMIL2* (encoding RLTPR; Fig. 2), which causes a CID with clinical features including hypogammaglobulinemia, poor humoral/Ag-specific immune responses, and few memory B cells (Lévy et al., 2023; Wang et al., 2016). Interestingly, RLTPR functions downstream of CD28, enabling CD4^+^ T cell activation and differentiation (Wang et al., 2016). Strikingly, cTfh cells, total and class-switched memory B cells, and levels of total and Ag-specific serum Ig are all intact in CD28-deficient humans (Béziat et al., 2021). Thus, this finding reveals that CD28 is dispensable for generating long-lived humoral immunity in humans and indicates fundamental CD28-independent roles for RLTPR in human immunity. These findings underscore the critical role of Tfh cells in mediating B cell differentiation during natural infection and vaccination, even when B cells are intrinsically functional, and highlight how IEI can identify key Tfh-dependent mechanisms regulating humoral immunity.

#### B cell defects due to inborn errors of NF-κB signaling

The NF-κB family of transcription factors regulate expression of >500 genes involved in immune cell proliferation, inflammation, development, and survival (Zhang et al., 2017). The canonical pathway (NF-κB1) is activated by many receptors including the BCR, Toll-like receptors, and CD40, while the non-canonical (NF-κB2) pathway is restricted to receptors that are typically expressed by B cells such as CD40, BAFF-R, and lymphotoxin-β receptor (LT-βR), and bind the TNF ligands CD40L, BAFF and APRIL, and LT-β, respectively (Fig. 4; Zhang et al., 2017). When activated, NF-κB1 (p105) and NF-κB2 (p100) are phosphorylated and cleaved to their respective active subunits (p50 and p52) that dimerize with other NF-κB members (RelA/p65, RelB, and c-Rel) and translocate to the nucleus. NF-κB signaling is restrained by inhibitory proteins (e.g., inhibitor of κB [IκBα]) that retain NF-κB1/2 in the cytoplasm, preventing their activation (Zhang et al., 2017; Fig. 4). Here, we review some IEIs due to germline mutations in genes encoding canonical and non-canonical NF-κB pathway proteins that likely contribute to disease via B cell–intrinsic mechanisms. Remarkably, most of these gene defects have little if any effect on B cell development in the BM, indicating that NF-κB signaling is largely redundant for B cell development.

*NF-κB1 deficiency.* NF-κB1/p50 haploinsufficiency, caused by heterozygous mutations in *NFKB1*, was initially discovered in 13 individuals from three kindreds with a diagnosis of CVID (Fliegauf et al., 2015; Fig. 3). Since then, >200 patients with *NFKB1* mutations have been identified that disrupt NF-κB1 expression, stability, phosphorylation, or nuclear transport (Kaustio et al., 2017; Li et al., 2021; Lorenzini et al., 2020; Tuijnenburg et al., 2018; Tuovinen et al., 2023). Most affected individuals present with late onset hypogammaglobulinemia, recurrent respiratory tract infections, lymphoproliferation, autoimmunity, B cell lymphopenia, and reductions in switched memory B cells (Kaustio et al., 2017; Li et al., 2021; Lorenzini et al., 2020; Tuijnenburg et al., 2018; Tuovinen et al., 2023). Impaired differentiation of patient B cells in vitro argues that *NFKB1* variants intrinsically disrupt B cell function (Tuijnenburg et al., 2018). Thus, NF-κB1 LOF compromises human B cell survival and/or differentiation, resulting in immune dysregulation (Fig. 4).

*IKKγ/NEMO deficiency.* IKKγ (NEMO, NF-*κB* essential modulator) encoded by *IKBKG* is a regulatory subunit of the kinase complex that phosphorylates IκBα leading to its degradation and subsequent activation and nuclear translocation of NF-κB1 (Zhang et al., 2017). Hemizygous hypomorphic *IKBKG* mutations cause XL anhidrotic ectodermal dysplasia with immunodeficiency (XL-EDA-ID; Döffinger et al., 2001; Jain et al., 2001). More than 90% of patients suffer from severe recurrent infections, and ∼60–80% exhibit hypogammaglobulinemia, impaired functional Ab responses, and reduced memory B cells (Hanson et al., 2008; Jain et al., 2004; Ma et al., 2015; Orange et al., 2004; Renner et al., 2021). The heterogeneity in clinical presentation of NEMO deficiency reflects the extreme variability in NEMO function due to different hypomorphic *IKBKG* variants (Hanson et al., 2008; Jain et al., 2004; Orange et al., 2004; Renner et al., 2021).

These clinical features of XL-EDA-ID are reminiscent of CD40L or CD40 deficiency (de la Morena, 2016; Notarangelo et al., 2006; Renner et al., 2021). This clinical overlap reflects a key role of NEMO in CD40/NF-κB1 signaling (Fig. 4). Indeed, CD40L-induced cRel activation, proliferation, and Ig CSR were dramatically disrupted in B cells with hypomorphic *IKBKG* mutations (Jain et al., 2001; Jain et al., 2004; Ma et al., 2016), indicating that B cell–intrinsic defects contribute to disease etiology due to NEMO deficiency. Furthermore, complete cRel deficiency prevented the generation of Ag-specific Abs, and reduced memory B cell formation and Ig CSR in vivo, as well as intrinsically abolished CD40L-induced B cell differentiation in vitro (Beaussant-Cohen et al., 2019; Lévy et al., 2021; Figs. 3 and 4). Thus, CD40L-mediated activation of the NF-κB1 pathway, involving NEMO, NF-κB1, and cRel, is critical for inducing B cell differentiation and establishing humoral immunity (Figs. 2 and 4). Notably, cTfh cells are also reduced in patients with hemizygous *IKBKG* or recessive *CREL* mutations (Lévy et al., 2021; Ma et al., 2015), which would further impact humoral immunity.

*CARD11/BCL10/MALT1 (CBM) complex.* The BCR is functionally linked to the NF-κB1 pathway by a multimeric complex comprising CARD11, BCL10, and MALT1 (Lu et al., 2018; Lu et al., 2019; Fig. 4). CARD11 is recruited to the BCR following ligation, where it is phosphorylated and undergoes conformational change enabling recruitment of BCL10 and MALT1, which constitutively associate with one another. The CBM complex then regulates activation of the canonical NF-κB1 pathway (Lu et al., 2018; Fig. 4). Recessive mutations in *CARD11* (8 patients; Greil et al., 2013; Lu et al., 2021; Nguyen et al., 2023a; Stepensky et al., 2013), *MALT1* (∼20 patients; Charbit-Henrion et al., 2017; Frizinsky et al., 2019; Jabara et al., 2013; Kutukculer et al., 2021; McKinnon et al., 2014; Punwani et al., 2015; Sonoda et al., 2021), or *BCL10* (4 patients; Al-Tamemi et al., 2022; Garcia-Solis et al., 2021; Torres et al., 2014; Van Den Rym et al., 2020) all result in a CID due to profound defects in lymphocyte differentiation (Lu et al., 2018; Lu et al., 2019). Deficiencies of these key components of BCR signaling did not disrupt B cell development per se, as circulating B cells were present in the patients in normal numbers. However, there was a marked defect in peripheral B cell development and differentiation, evidenced by an accumulation of immature transitional B cells, a complete absence of memory B cells and hypogammaglobulinemia (Fig. 1). Thus, canonical NF-κB1 signaling mediated via the CBM complex is redundant for B cell development but critical for the generation of a pool of naive B cells (Lu et al., 2018; Lu et al., 2019). This likely reflects a B cell–intrinsic requirement for CARD11, BCL10, and MALT1 during the final stages of B cell development that occur in peripheral lymphoid organs, while the paucity of memory B cells may also arise from impaired CD4^+^ T cell help as these proteins are also expressed in T cells and have similar functions in TCR signaling (Lu et al., 2018; Lu et al., 2019).

Monoallelic activating variants in *CARD11* have also been identified in individuals with childhood-onset polyclonal B cell lymphocytosis. The first clinical description of this condition was actually reported in 1971 (Darte et al., 1971), and it was solved genetically in 2012 when the two daughters of the index case also presented with these clinical features and were investigated by next-generation sequencing (Snow et al., 2012; Fig. 3). Since then, ∼30 cases of *CARD11* GOF have been described, with clinical features including B cell lymphocytosis, splenomegaly, lymphadenopathy, and recurrent bacterial (100% of cases) and viral (30–45% cases) infections (Lu et al., 2018; Shields et al., 2020). Laboratory investigations revealed that the vast increase in peripheral blood B cell numbers was due to an expansion of transitional and naive B cells, but a paucity of total and Ig class-switched memory B cells (Arjunaraja et al., 2017; Figs. 1 and 2). Consistent with this block in peripheral B cell maturation, and recurrent infections, serum Ig levels and Ab responses to polysaccharide or protein vaccines were reduced in many patients with *CARD11* GOF (Lu et al., 2018; Lu et al., 2019). *CARD11* GOF B cells were unable to differentiate into PCs following in vitro stimulation, establishing that aberrant CARD11 function disrupts B cell differentiation in a cell intrinsic manner (Arjunaraja et al., 2017). Mechanistically, GOF variants result in spontaneous activation of CARD11 and CBM formation, and subsequent constitutive NF-κB1 signaling without requiring BCR engagement (Lu et al., 2018; Lu et al., 2019). Thus, elevated basal NF-κB1 signaling leads to B cell lymphoproliferation and impaired humoral immunity.

*NF-κB2 deficiency.* Inactivating mutations in *NFKB2* encoding NF-κB2 (p100/p52) were initially identified in individuals who presented with hypogammaglobulinemia with or without endocrine or autoimmune features (Brue et al., 2014; Chen et al., 2013; Lee et al., 2014; Liu et al., 2014; Fig. 3). Most variants target the C-terminus of NF-κB2/p100 (Klemann et al., 2019) which contains critical phosphorylation sites necessary for processing p100 to the active p52 form. Thus, nuclear translocation of p52 was impeded in CD40L-stimulated B cells lines from patients (Chen et al., 2013). Typical clinical features of NFKB2 deficiency are early onset hypogammaglobulinemia, recurrent respiratory infections, poor Ab responses to vaccines, autoimmunity, and adrenocorticotropic deficiency (Brue et al., 2014; Chen et al., 2013; Klemann et al., 2019; Lee et al., 2014; Liu et al., 2014). Patients with *NFKB2* deficiency exhibit normal/low B cell numbers, or even complete B cell deficiency, possibly due to progressive B cell lymphopenia (Brue et al., 2014; Chen et al., 2013; Klemann et al., 2019; Lee et al., 2014; Liu et al., 2014). Interestingly, analysis of BM from one patient revealed a partial block in B cell development at the pre-B stage (Lougaris et al., 2019; Fig. 1). When peripheral B cells are present, severe reductions in memory B cells have been observed (Brue et al., 2014; Chen et al., 2013; Klemann et al., 2019; Lee et al., 2014; Liu et al., 2014; Fig. 2). Notably, the T cell compartment is generally less affected by *NFKB2* variants than B cells; however, cTfh cells are generally lower in these patients compared to healthy donors (Klemann et al., 2019; Fig. 2). Thus, NF-κB2 signaling, likely downstream of CD40 and BAFF-R, is critical for intact human B cell development, differentiation, and function (Fig. 4).

The importance of non-canonical NF-κB2 signaling is further highlighted by the clinical phenotypes of individuals with biallelic variants in either *MAP3K14* (encoding NF-κB–inducing kinase [NIK]; Willmann et al., 2014), which acts upstream of NF-κB2, or *RELB* (Sharfe et al., 2015), which dimerizes with NF-κB2 (Fig. 4). Deficiency of NIK or RelB impacts peripheral B cell maturation, evidenced by an accumulation of transitional B cells, hypogammaglobulinemia, and dramatic reductions in circulating memory B cells, as well as impaired CSR and Ab responses to infections and vaccines (Sharfe et al., 2015; Willmann et al., 2014). B cells from these patients exhibit poor responses to stimulation with BAFF or CD40L in vitro, including reduced NF-κB2 signaling, survival, upregulation of activation markers, and Ig class switching (Sharfe et al., 2015; Willmann et al., 2014), indicating B cell–intrinsic defects contribute to the clinical features of NIK deficiency or RelB deficiency. NIK-deficient B cells also express reduced levels of ICOS-L and fail to further upregulate its expression in response to CD40L signaling (Willmann et al., 2014). Consistent with this, and similar to individuals with mutations in *CD40LG*, *ICOS*, *ICOSLG*, or *NFKB2* (Tangye and Ma, 2021), cTfh cells are reduced in NIK-deficient individuals (Sharfe et al., 2015; Willmann et al., 2014; Fig. 2). While the Tfh deficit may be secondary to defective B cell function, recessive mutations in *MAP3K14* or *RELB* do significantly affect T cells, indicating that the clinical features of these IEI likely result from impaired effector functions of many immune lineages, rather than being disease-causing by predominantly impacting B cells (Sharfe et al., 2015; Willmann et al., 2014).

*Impaired signaling via BAFF and APRIL.* Two siblings have been reported with homozygous mutations in *TNFRSF13C*, encoding BAFF-R which signals predominantly via the NF-κB2 pathway (Warnatz et al., 2009; Fig. 4). Laboratory findings of these individuals partially overlapped with NIK deficiency or RelB deficiency, including B cell lymphopenia, reduced serum IgM and IgG (but normal levels of IgA), poor responses to pneumococcal polysaccharides vaccines, an accumulation of transitional B cells and corresponding paucity of total and class-switched memory B cells in peripheral blood. However, B cell development in the BM, SHM in peripheral blood B cells, Ab responses to tetanus vaccination, as well as the distribution of T cell subsets were unaffected by BAFF-R deficiency (Warnatz et al., 2009). Thus, impaired BAFF-R signaling results in a selective block in B cell maturation at the transitional → naive stages (Figs. 1 and 4), thereby significantly impacting the final stages of B cell development that occur in the periphery and manifesting as B cell deficiency and impaired T-independent immune responses. This is consistent with BAFF functioning as a survival factor for human and murine B cells (Tangye et al., 2006). Interestingly, the index case was diagnosed with CVID late in life (aged 57 yr) following a history of recurrent infections (Warnatz et al., 2009), while recessive *TNFRSF13C* mutations were clinically silent in the sibling, suggesting compensatory pathways may overcome BAFF-R deficiency. Despite this, the discovery of individuals with BAFF-R deficiency has shed light on mechanisms underlying different IEIs affecting NF-κB. Thus, as BAFF-R almost exclusively signals via the NF-κB2 pathway (Tangye et al., 2006), impaired responsiveness to BAFF likely explains defective peripheral B cell maturation in individuals with NIK or RelB deficiency (Sharfe et al., 2015; Willmann et al., 2014), while an inability to respond to CD40L and other TNFR ligands would contribute to the more severe clinical phenotype of these patients compared to those with BAFF-R deficiency.

BAFF can also bind the TNFR-SF members TACI and BCMA; furthermore, the related TNF ligand APRIL binds TACI and BCMA, but not BAFF-R (Tangye et al., 2006; Pieper et al., 2013). Heterozygous variants have been identified in *TNFRSF13B*, encoding TACI, in 5–10% of CVID patients (Bogaert et al., 2016; Pieper et al., 2013). However, the same *TNFRSF13B* variants are present in 1–2% of healthy donors, and the penetrance of a CVID phenotype due to *TNFRSF13B* variants is incredibly variable (Bogaert et al., 2016). Thus, *TNFRSF13B* variants are likely to be disease-modifying alleles rather than pathogenic. Recently, the first case of APRIL deficiency due to homozygous nonsense variants in *TNFSF13*, encoding APRIL, was reported in an individual diagnosed with adult-onset CVID (Yeh et al., 2020; Fig. 3). The patient presented with hypogammaglobulinemia (low IgM, IgG; very low IgA) and mild infections that were controlled with Ab replacement. While numbers of peripheral B cells, including IgG^+^ and IgA^+^ memory B cells, the BCR repertoire and levels of SHM were similar to healthy donors, circulating PCs were reduced 10-fold in the absence of APRIL (Yeh et al., 2020; Fig. 4). Although it is difficult to draw too many conclusions from a single case, these findings confirm a likely role for APRIL in maintaining PC survival and serum Ig levels (Fig. 2). However, like BAFF-R deficiency, the clinical phenotype of APRIL deficiency was late onset and relatively mild, suggesting modest, albeit important, roles for the BAFF/APRIL system in maintaining humoral immunity in humans. Indeed, the findings that IgA levels are particularly low in APRIL deficiency (Yeh et al., 2020), but IgA^+^CD27^−^ B cells are present at normal numbers in CD40L deficiency (van Zelm et al., 2014), suggests APRIL predominantly regulates IgA class-switching and production by human PC.

#### Dysregulated PI3K signaling causes immune dysregulation

Biallelic mutations in *PIK3R1* or *PIK3CD* established the fundamental requirement for PI3K signaling in B cell development (Fig. 1; Conley et al., 2012; Sogkas et al., 2018; Tang et al., 2017). The discovery of heterozygous GOF *PIK3CD* (p110δ catalytic subunit; Angulo et al., 2013; Lucas et al., 2014a) or LOF *PIK3R1* (p85 regulatory subunit) mutations (Deau et al., 2014; Lucas et al., 2014b)—which both increase PI3K activation—revealed a key role for balanced PI3K signaling in B cell development and differentiation (Fig. 3). Affected individuals present with recurrent respiratory infections, poor immune responses to vaccines, hypogammaglobulinemia with occasional increased levels of serum IgM, susceptibility to viral infections, lymphadenopathy, autoimmunity, and B cell malignancy (Angulo et al., 2013; Deau et al., 2014; Lucas et al., 2014a; Lucas et al., 2014b; Tangye et al., 2019). These conditions are termed activated PI3K-δ syndrome (APDS1: *PIK3CD*; APDS2: *PIK3R1*).

*PIK3CD* GOF and *PIK3R1* LOF variants cause a partial block at the pro/pre-B cell stage, resulting in few mature B cells in the BM (Fig. 1). APDS patients also have low numbers of peripheral B cells, significantly increased frequencies of transitional cells, and reductions in naive and memory cells (Figs. 1 and 2; Avery et al., 2018; Dulau Florea et al., 2017; Nguyen et al., 2023b). In vitro assessment revealed intrinsic differentiation defects, evidenced by impaired CSR and Ig secretion by *PIK3CD* GOF/*PIK3R1* LOF B cells (Avery et al., 2018; Nguyen et al., 2023b), which likely contribute to poor humoral immune responses in APDS. Importantly, some in vitro B cell defects could be overcome by pharmacological attenuation of p110δ (Avery et al., 2018; Nguyen et al., 2023b), thus establishing that hyperactive PI3K causes this disorder. Indeed, the p110δ-specific inhibitor leniolisib corrects aberrant B cell phenotypes in vivo and substantially improves clinical features of APDS (Rao et al., 2022).

PI3K is directly regulated by the lipid phosphatases PTEN and SHIP (Tangye et al., 2019). Heterozygous *PTEN* mutations are associated with PTEN harmatoma tumor syndromes and neurodevelopmental delay (Tangye et al., 2019). However, PTEN deficiency has recently also been associated with an APDS-like clinical phenotype, including autoimmunity, lymphoid hyperplasia, hypogammaglobulinemia, reduced responses to vaccinations and recurrent infections, CD4^+^ T cell lymphopenia, transitional B cell accumulation, and reduced memory B cells (Browning et al., 2015; Chen et al., 2017; Driessen et al., 2016; Tsujita et al., 2016). The underlying biochemical defect in these patients is likely to be similar to *PIK3CD* GOF and *PIK3R1* LOF individuals, as PTEN mutations also result in enhanced PI3K signaling (Browning et al., 2015; Tsujita et al., 2016). However, more detailed analysis of immune defects in individuals with *PTEN* mutations is required to determine whether the severity and penetrance of their clinical feature are as dramatic as those observed in individuals with *PIK3CD* GOF or *PIK3R1* LOF mutations.

#### IL-21R/STAT3 signaling in B cells is required for generating long-lived humoral immunity and memory

Over the past four decades, studies have established that many cytokines induce human B cell differentiation in vitro (Moens and Tangye, 2014). However, assessment of humoral immunity and memory in IEIs has revealed that IL-21 is the fundamental cytokine driving human B cell differentiation in vivo. IL-21R comprises the IL-21R chain associated with the common γ chain (γc). IL-21/IL-21R signaling activates STAT1, STAT3, and STAT5, and induces lymphocyte proliferation, differentiation, and effector function (Leonard, 2001; Tangye and Ma, 2020). A key regulator of STAT3 expression and activation is the transcription factor ZNF341 (Fig. 4; Béziat et al., 2018; Frey-Jakobs et al., 2018). Strikingly, IL-21 induces all the intrinsic molecular requirements necessary for GC formation and B cell differentiation: proliferation, *AICDA* and *IRF4* for CSR, *BCL6* to form GCs, and *PRDM1* (Blimp-1), *XBP1*, and *IRF4* to mediate PC formation (Avery et al., 2010; Deenick et al., 2013; Dvorscek et al., 2022; Quast et al., 2022; Tangye and Ma, 2020).

DN *STAT3* (Holland et al., 2007; Minegishi et al., 2007) or biallelic LOF *ZNF341* (Béziat et al., 2018; Frey-Jakobs et al., 2018) mutations cause recurrent bacterial and fungal infections, impaired functional Ab responses and few circulating memory B cells (Avery et al., 2010; Béziat et al., 2018; Chandesris et al., 2012; Frey-Jakobs et al., 2018; Leung et al., 1988; Sheerin and Buckley, 1991). *STAT3* DN or *ZNF341* LOF naive B cells fail to differentiate in vitro into Ab-secreting cells in response to IL-21 (Avery et al., 2010; Béziat et al., 2018; Deenick et al., 2013). Interestingly, biallelic variants in *IL21* or *IL21R* cause recurrent infections, hypogammaglobulinemia, and reductions in Ag-specific Ab and total and class-switched memory B cells (Cagdas et al., 2021; Kotlarz et al., 2013; Ma et al., 2015). Not surprisingly, mutations in *IL21R* or *IL2RG* (γc) prevent B cell responses to IL-21 (Cagdas et al., 2021; Kotlarz et al., 2013; Recher et al., 2011; Tangye et al., 2023). Furthermore, memory B cell formation, humoral immunity and hypogammaglobulinemia were not restored by HSC transplant of patients with *IL2RG* variants who retained their own γc-deficient B cells but engrafted donor (*IL2RG* WT) T cells after transplant (Recher et al., 2011).

Inborn errors affecting IL-10/IL-10R, IL-12/IL-12R, IL-23R, IL-6R, IFNγ/IFNγR, type I IFN/IFNαR, or IL-17/IL-17R have a mild—if any—impact on humoral immunity, as determined by quantifying basal levels of memory B cells and serum Ig, as well as of Ag-specific memory B cells and Abs following natural infection and/or vaccination (Beziat et al., 2020; Chen et al., 2021; Kotlarz et al., 2012; Ma et al., 2015; Philippot et al., 2023; Schwerd et al., 2017; Shahin et al., 2019; Sharifinejad et al., 2022; Sokal et al., 2023; Spencer et al., 2019). Consistent with this, memory B cell formation and serum Ig levels are within or exceed normal ranges in patients with inactivating mutations in *STAT1*, which is activated by several of these cytokines (Avery et al., 2010; Deenick et al., 2013; Ma et al., 2015). Combined, these findings revealed that IL-21R:γc/STAT3/ZNF341 signaling in B cells is the predominant cytokine pathway responsible for inducing and maintaining robust long-lived humoral immunity in humans (Fig. 2).

A novel IEI was recently reported in seven individuals from six families due to a recurrent heterozygous *IRF4* variant (IRF4^T95R^; International Consortium et al., 2023). These patients had opportunistic infections, agammaglobulinemia, and a severe deficiency of memory B cells and PC. Patient naive B cells were unable to undergo CSR and PC differentiation in vitro, establishing that B cell–intrinsic effects of IRF4^T95R^ contributed to their humoral defects (International Consortium et al., 2023). IL-21 induces IRF4 expression in activated B cells (Deenick et al., 2013; Luo et al., 2023), and IRF4 upregulates AID and promotes CSR and PC generation in vivo in an IL-21–dependent manner (Klein et al., 2006; Luo et al., 2023). Thus, the overlap in clinical features of patients with IL-21R deficiency or IRF4^T95R^ may reflect the IL-21/IRF4 axis being a major regulator of late B cell differentiation (Figs. 2 and 4). Notably, cTfh cells are also reduced in patients with the IRF4^T95R^ variant (International Consortium et al., 2023), confirming a role for IRF in Tfh generation (Fig. 3).

### Humans are not mice

Much of our understanding of immunology has been derived from studies performed in mouse models. This, of course, is an entirely suitable approach to address biological and physiological phenomena—however, it is often assumed that mechanisms underpinning processes in one species also apply in another. And while this can indeed be true, it isn’t always. Thus, it is worth highlighting that the study of IEI has revealed fundamental differences between the molecular requirements for B cell development and differentiation between mice and humans. Examples include:

• The block in B cell development in mice lacking *Btk* occurs later than in humans with XLA; this results in a milder reduction in numbers of peripheral B cells and levels of serum Ig (Conley et al., 2009);
• *Baffr* deficiency in mice impedes B cell development at the early transitional stage, resulting in dramatic reductions in B cell numbers and an immunodeficient state that is not recapitulated in humans with *TNFRSF13B* mutations (Bogaert et al., 2016; Pieper et al., 2013; Warnatz et al., 2009);
• Unlike mice lacking IL-7/IL-7R signaling, B cells develop in humans with biallelic variants in *IL7RA* (Giliani et al., 2005; Puel and Leonard, 2000), suggesting human B cell development is independent of IL-7.

These examples are not simply important from a biological perspective but also clinically, because therapies have been developed that target BTK (ibrutinib) or BAFF (belilumab) to treat B cell diseases (Paley et al., 2017), or utilize IL-7 as an immunostimulatory adjuvant therapy for treating cancer, HIV, or immune reconstitution (Morre and Beq, 2012). Thus, understanding the roles of these molecules and pathways in humans is critical to ensure maximum efficacy of targeted therapeutics in treating human immune dyscrasias.

## Conclusion

It has been over 70 yr since Bruton first reported agammaglobulinemia, and 30 yr since the discovery that *BTK* mutations cause XLA. By studying inborn errors affecting humoral immunity, the past three decades has seen an exponential increase in our understanding of fundamental molecular, biochemical, and cellular requirements underpinning the development and differentiation of human B cells. These studies have identified non-redundant intrinsic signaling pathways, transcriptional networks, and cognate cell–cell interactions that enable the generation of immunocompetent naive B cells that undergo Ag-specific expansion and affinity maturation to generate memory cells and PC to ensure long-lived humoral immunity and host defense against infectious disease. Discoveries from IEI have also impacted our understanding of mechanisms causing immune dysregulation (autoimmunity, allergy, and malignancy), enabled therapeutic interventions for B cell–mediated immunopathologies, and have the potential to improve the design of future vaccinations strategies. However, as starkly revealed by the COVID-19 pandemic, we still have much to learn about long-lived humoral immunity and maximizing vaccine efficacy. No doubt these and other unknowns will be solved in part by future discoveries of novel inborn errors affecting human B cells.
